# The perspective of patients with early rheumatoid arthritis on the journey from symptom onset until referral to a rheumatologist

**DOI:** 10.1093/rap/rkz035

**Published:** 2019-08-30

**Authors:** Diederik De Cock, Kristien Van der Elst, Veerle Stouten, Donna Peerboom, Johan Joly, Rene Westhovens, Patrick Verschueren

**Affiliations:** Department of Development and Regeneration, Skeletal Biology and Engineering Research Center, University of Leuven, Leuven, Belgium

**Keywords:** rheumatoid arthritis, patient perspective, patient trajectory, treatment delay, general practitioner, referral pathway

## Abstract

**Objective:**

Timely treatment of patients with early RA (ERA) favours a beneficial disease outcome. However, individuals often delay their contact with a health-care professional (HCP) after ERA-related symptom onset. The aim of this study was to investigate the perspective of patients on the journey of a patient from RA symptom onset until referral to a specialist.

**Methods:**

A subgroup of patients with ERA from the Care in ERA (CareRA) trial were interviewed retrospectively to discuss their initial ERA-related experiences preceding diagnosis, using a bespoke assessment form. The first section of the form focused on initial symptoms and help-seeking behaviour by the patients. The second part probed the actions of the HCPs consulted. Additional notes derived from the patient stories were analysed thematically.

**Results:**

Among 94 patients, pain (97%), swelling (73%) and stiffness (52%), typically in multiple joints, were reported as initial ERA symptoms. The general practitioner (GP) was generally the first HCP to be contacted (87%). Frequently reported reasons to visit an HCP were intense pain (90.4%) and difficulties in performing daily activities (69%). In 44.1% of patients, the HCP suspected ERA at the first visit. Approximately 25% of patients needed more than five visits before detection of ERA. GPs mainly referred patients to rheumatologists (71%). Thematic analysis uncovered that multiple HCPs were often involved in the journey to RA detection and referral.

**Conclusion:**

Pain is the most commonly reported initial symptom of ERA and the main reason to visit an HCP, usually a GP. These GPs play a pivotal role in early detection and correct referral. Furthermore, the journey of a patient seems complex, often with multiple HCPs being involved.

**Key messages**

- Excess pain seems to be the most important trigger for seeking help in persons susceptible to RA.
- General practitioners play a pivotal role in RA detection but are hindered by different initial symptoms of RA.
- Multiple visits to the general practitioner and longer treatment delays impact RA patients’ perceptions and health behaviours.

## Introduction

International guidelines recommend the treatment of patients with RA early, intensively and to target [1–3]. Initiation of RA treatment within 3–4 months after symptom onset is believed to favour long-term disease control and prevent structural damage of the joints [4, 5]. A study by Van Nies *et al.* [6] quantified this time frame of the window of opportunity, with the optimal time for treatment initiation being <14–19 weeks after symptom onset. However, timely treatment appears challenging, because treatment delay, defined as the time elapsed between symptom onset and initiation of treatment, is still too long for the majority of individuals in many countries based on this window of opportunity of <14–19 weeks [7–17].

The patient-related delay, defined as the time between symptom onset and the first visit to a health-care professional (HCP) regarding these symptoms, seems to contribute to a large extent to the overall delay [15, 18]. A person’s help-seeking behaviour is influenced not only by clinical disease features, but also by psychosocial factors, such as illness perception, coping and social interactions [19–23].

A qualitative study by Stack *et al.* [24] demonstrated that symptoms and symptom patterns of individuals in their earliest stages of RA are very heterogeneous, including joint pain, psychological distress, muscle cramps, abnormal skin sensations, stiffness, loss of motor control, weakness, fatigue and difficulties in sleeping. However, there is still limited evidence about whether these factors contribute to a person’s decision to seek medical help and the journey that patients follow from symptom onset until referral. Only one case–control study in a large general practice register by Muller *et al.* [25] described the symptoms of patients before RA diagnosis. Patients who went on to develop RA had more general practitioner (GP) visits in the months before the final diagnosis with, in general, more joint symptoms *vs* control patients. A cohort study in a small general practice sample also evaluated the patient’s signs and symptoms and the additional investigations performed by the GP [26]. That study showed that GPs focused mainly on classical signs of inflammation.

Hence, the aim of the present study was to add to current knowledge about the patient-related delay in diagnosing RA patients with early RA (ERA) experienced the period between symptom onset and referral by a HCP.

## Methods

In this cross-sectional, single-centre study, we collected data from patients with early RA who participated in the Flemish Care in early RA (CareRA) trial [27–29], followed at the University Hospitals Leuven. Information on symptom onset and referral date was collected from a previous study by our research group [15]. The Ethics Committee of the University Hospitals Leuven approved the CareRA study (EudraCT number 2008–007225-39). All patients gave written informed consent.

Between November 2012 and August 2014, participants were interviewed about their initial RA-related symptoms and journey towards referral by K.E. (clinical nurse) or V.S. (clinical research assistant) during an outpatient visit, using a bespoke assessment form about their initial RA-related symptoms. This assessment form was based on questions routinely asked of newly diagnosed patients by rheumatologists and study nurses at the first clinical visit. For this study, these questions were structured into a standardized assessment form.

### Instrument

The bespoke assessment form (in Dutch and translated into English) is available as supplementary material, available at *Rheumatology Advances in Practice* online. The first section of the assessment form focused, with eight questions, on patients’ perceptions of initial symptoms and their help-seeking behaviour.

1. Date of symptom onset: the time elapsed between the date of symptom onset and CareRA trial inclusion or completion of the assessment form defined symptom duration and recall period.
2. Initial symptoms: predefined symptom categories included pain, swelling, stiffness in the joints, fatigue, morning stiffness and a free text category. Pain, swelling and stiffness in the joints could be specified in one or multiple joints and in specific areas where these symptoms arose (i.e. the fingers, hands and wrists; toes, feet and ankles; knees; shoulders and elbows; free text).
3. Date of first musculoskeletal problems, if first symptoms were not pain, swelling or stiffness in the joints.
4. Date of persistent swelling if different from the symptom onset date.
5. Concurrent events shortly before or at symptom onset (i.e. injury, pregnancy, surgery, infection, vaccination or free text).
6. Which HCP patients visited first regarding their first rheumatic symptoms (i.e. GP, rheumatologist, physiotherapist, orthopaedic surgeon, neurologist, osteopath or free text).
7. Date of first visit to an HCP.
8. Reasons to seek medical help (i.e. having difficulties performing daily activities; intense pain; severe stiffness; remarkable swelling; difficulties performing professional activities; free text).

The second section of the form probed patients’ perspectives, in four questions, about the actions that the consulted HCP(s) undertook until referral to a specialist.

1. Did the HCP recognize at the first visit their symptoms as possibly RA related (yes/no)? If ‘No’, patients could indicate which other disorders were considered by the HCP, and they were asked for the number of return visits (one, two, three, four, five or more than five) to this HCP before RA was suspected.
2. Diagnostic and therapeutic steps undertaken by the HCP before referral (i.e. blood analysis; radiographic examinations; prescription of pain medication; wait-and-see approach; advice on lifestyle; free text).
3. What type of specialist patients were initially referred to by their first consulted HCP (i.e. rheumatologist; physiotherapist; orthopaedic surgeon; neurologist, osteopath; free text).
4. Date of referral to the treating rheumatologist.

### Data analysis

The results are presented descriptively as the mean (s.d.), median (interquartile range) or percentages. Analyses were performed using SPSS v.22.0.

Patient information not fitting the (predefined) answers in the assessment form was written down as additional notes and sketches. A sketch could be, for example, a simple figure with arrows depicting the journey of the patient from HCP to HCP. These comments and sketches were appraised by thematic analysis. This additional patient-provided information was independently read, coded and categorized by D.C., K.E. and D.P. Thereafter, themes were formed by consensus.

## Results

From 106 eligible patients, data of 12 patients could not be collected owing to death (*n* = 2), loss to follow-up (*n* = 9) and trial consent withdrawal (*n* = 1). Table 1 displays the characteristics of the 94 participants in the present study at recruitment in the CareRA trial. Dates from 86 participants were available for symptom duration and recall period. These mean (s.d.) time intervals were 153.5 (115.8) and 105.0 (114.5) weeks, respectively.

**Table 1:** Characteristics of the study population at the time of recruitment into the CareRA trial

Variable	Total study participants (*n* = 94)
Age, mean (s.d.), years	52.2 (14.2)
Women, *n* (%)	70 (75)
BMI (kg/m²), mean (s.d.)	26.6 (4.4)
Current smoker, *n* (%)	24 (23)
RF positive, *n* (%)	58 (62)
ACPA positive, *n* (%)	69 (73)
PGA (0–100), mean (s.d.)	57.5 (24.4)
Pain (0–100), mean (s.d.)	58.7 (22.6)
Fatigue (0–100), mean (s.d.)	51.1 (23.3)
PhGA (0–100), mean (s.d.)	49.7 (18.0)
Total 66-SJC, mean (s.d.)	12.5 (6.7)
Total 68-TJC, mean (s.d.)	17.3 (10.6)
CRP (mg/l), mean (s.d.)	20.2 (33.5)
ESR (mm/h), mean (s.d.)	34.8 (25.4)
DAS28CRP (0–9.4), mean (s.d.)	4.9 (1.3)
HAQ (0–3), mean (s.d.)	1.0 (0.8)

Results are given as the mean (s.d.) or as the number (percentage).

CareRA: care in early RA; DAS28CRP: 28-joint DAS calculated with CRP; PGA: patient global assessment; PhGA: physician global assessment; SJC: swollen joint count; TJC: tender joint count.

### Initial symptoms

Most of the participants (97%, 91 of 94) reported pain at symptom onset, followed by swelling (73.4%, 69 of 94), stiffness in the joints (52%, 48 of 94), morning stiffness (33%, 31 of 94) and fatigue (21%, 20 of 94). Other types of symptoms were experienced by 14% (13 of 94) of the patients, including joint redness (*n* = 4), loss of strength (*n* = 4), joint inflammation (*n* = 2), weight loss (*n* = 1), emotional distress (*n* = 1) and stiff legs (*n* = 1).

Multiple joints were painful at symptom onset in 79% (65 of 82) of the participants. Pain at symptom onset was experienced by 71% (64 of 90) of patients in fingers, hands and/or wrists; by 42% (38 of 90) of patients in toes, feet and/or ankles; by 18% (16 of 90) in the knees and by 19% (17 of 90) in shoulders or elbows. Two patients indicated painful legs and one a painful hip.

Multiple joints were swollen at symptom onset in 72% (43 of 60) of the patients. A total of 76% (51 of 67) indicated swelling in the fingers, hands and/or wrists at symptom onset. Swelling that involved toes, feet and/or ankles was reported by 34% (23 of 67). The knees were swollen in 12% (8 of 67) and the shoulders or elbows in 10% (7 of 67) of patients. One patient mentioned swollen legs.

Multiple joints were stiff at symptom onset in 89% (39 of 44) of patients. A total of 83% (39 of 47) reported stiff fingers, hands and/or wrists, whereas 40% (19 of 47) reported stiff toes, feet and/or ankles. Stiff knees and shoulders or elbows were reported in 17% (8 of 47) and 15% (7 of 47) of patients. Two patients mentioned stiff legs.

Only five and eight patients mentioned first musculoskeletal problems and first persistent swelling as not present at initial symptom onset, respectively.

### Concurrent events at symptom onset

The majority (68%; 64 of 94) of patients reported no concurrent events shortly before or at symptom onset. Two patients reported having undergone surgery, one patient reported an injury, one patient attributed pregnancy to symptom onset and one patient vaccination. Other types of events attributed to disease onset were given in free text by 24 patients, including emotional distress (*n* = 8), physical distress (*n* = 6), co-morbidities (*n* = 4), bite/sting accident (*n* = 3), a change in medication (*n* = 2) and a holiday (*n* = 1).

### First consulted HCP owing to rheumatic complaints

General practitioners were by far the first contacted HCP (87%, 77 of 89), followed by rheumatologists (2%, 2 of 89), orthopaedic surgeons (2%, 2 of 89), neurologists (2%, 2 of 89) and physiotherapists (2%, 2 of 89). Other HCPs visited were mentioned by 7% (6 of 89) of patients, including three emergency physicians, one cardiologist, one ophthalmologist and one podiatrist. Two patients provided two answers for this question.

### Reason(s) for consulting the first HCP

The most common reason to seek medical help was experiencing intense pain (90%, 85 of 94). Having had problems with activities of daily living was indicated by 69% (65 of 94) of the patients, a remarkable swelling by 33% (31 of 94), too severe stiffness by 23% (22 of 94), and problems during professional activities by 19% (18 of 94). Nine patients provided other reasons to consult a HCP: three mentioned the presence of RA in the family, two reported walking difficulties, one felt sick, one felt tired/melancholic, one had “the same pain for too long” and one had a check-up for stomach complaints.

### Suspicion of RA by the first contacted HCP

According to 44% (41 of 93) of the patients, RA was immediately recognized as a possible cause of the symptoms experienced. Table 2 shows other disorders apart from RA considered by the first contacted HCP at first visit.

**Table 2:** Other disorders considered by the health-care professional apart from RA

Category of other disorders considered	Specification of disorders
Inflammatory musculoskeletal disorders (*n*=10)	Tenosynovitis (*n*=2)
	Local synovitis fingers/wrist (*n*=2)
	Bursitis (*n*=1)
	Further unspecified (*n*=5)
Structural/functional musculoskeletal disorders (*n*=15)	OA (*n*=2)
	Meniscus (*n*=2)
	Back problems (*n*=1)
	Frozen shoulder (*n*=1)
	Myalgias (*n*=1)
	Neck problems (*n*=1)
	Subluxation of metatarsophalangeal joints (*n*=1)
	Rotator cuff problem (*n*=1)
	Further unspecified (*n*=5)
Other musculoskeletal disorders (*n*=2)	Rheumatism (*n*=2)
Physical overburdening (*n*=6)	Further unspecified (*n*=6)
Neurological disorders (*n*=5)	Carpal tunnel syndrome (*n*=2)
	Multiple sclerosis (*n*=1)
	Further unspecified (*n*=2)
Other (*n*=8)	Chilblains (*n*=2)
	Thromboembolism (*n*=1)
	Allergic reaction (*n*=1)
	Cyst on left hand (*n*=1)
	Infection from tick bite (*n*=1)
	Lung infection (*n*=1)
	Related to emotions (*n*=1)
Health-care professional did not know (*n*=3)	Further unspecified (*n*=3)
Patient did not remember (*n*=1)	Further unspecified (*n*=1)

### Number of additional visits required before RA was suspected

**Fig. 1:**
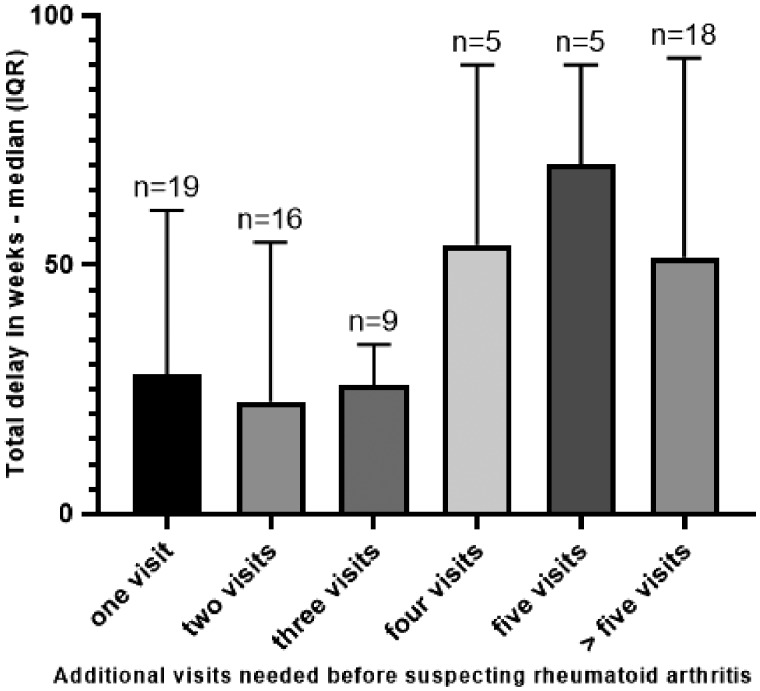
Number of additional visits before early RA was suspected with respect to the total treatment delay

Many patients needed several consultations before the HCP suspected RA. After the initial visit, 26% (19 of 72) of the patients required one more visit, 22% (16 of 72) needed two additional visits, 13% (9 of 72) needed three additional visits, 7% (5 of 72) needed four additional visits, 7% (5 of 72) needed five visits, and 25% (18 of 72) needed five more visits before probable RA was suspected (Fig. 1).

### Action undertaken by the consulted HCP

The most frequently reported diagnostic procedure was a blood test in 78% (68 of 87) of the patients, followed by radiography (15%, 13 of 87). Prescription of pain medication was done in 61% (53 of 87) of patients. Eight patients mentioned the prescription of medication such as cortisone or muscle relaxants, five reported advice on supportive devices such as splints and arch supports, one remembered advice on warmth application, and one drainage of the knee. Two patients did not remember any action taken by their HCP.

### To whom the patient was referred by the first consulted HCP

According to the participants, the first consulted HCP referred them to a rheumatologist (71%, 59 of 83), an orthopaedic surgeon (10%, 8 of 83), a physiotherapist (2%, 2 of 83) or a neurologist (1%, 1 of 83). Seven patients reported being referred to other HCPs: two emergency physicians, one acupuncturist, one infectious diseases physician, one nuclear medicine physician, one rehabilitation physician and one trauma surgeon. Five patients went to another HCP on their own initiative after visiting the first HCP: four patients went to a rheumatologist and one to a sports doctor. One patient could not remember.

### Thematic analysis of additional notes and sketches

While completing the assessment form, 88 of 94 patients mentioned information that could not be categorized under the predefined questions. This additional information was grouped into three themes: (a) familiarity with RA; (b) coping with the first RA-related symptoms; and (c) complexity of the referral pathway.

#### Familiarity with RA

In those patients who had family members with a rheumatic disease, RA seemed to be recognized promptly by the HCP, and they seemed to require fewer visits before referral to a rheumatologist. Some patients indicated that they had informal interactions with health professionals among their friends and relatives, such as doctors or nurses among their family members, helping them in seeking help for their symptoms. Patients who had knowledge about RA reported having scheduled an appointment with a rheumatologist at their own initiative. Furthermore, patients being more familiar with RA reported having asked their HCP whether their symptoms could be RA related and having asked for a blood test for RA.

#### Coping with the first RA-related symptoms

Many patients described episodic symptom onset. This resulted in an abundant reporting of supportive devices, such as inlay soles, plasters and support bandages, during waxing and waning periods of initial complaints.

#### Complexity of the referral pathway

In about one-third of the 88 patients, the journey from symptom onset to referral included the GP as the first-consulted HCP who suggested a referral to a rheumatologist. However, many patients mentioned having consulted a range of different HCPs before being referred to a rheumatologist. Patients who had family members with a rheumatic disease appeared to have a less complex referral pathway. Some patients mentioned going to the emergency department owing to the severity of symptoms, mostly while waiting for a first appointment with a rheumatologist. Even after referral, several patients indicated that they had visited several rheumatologists. Furthermore, some felt misunderstood or not listened to by their HCP or rheumatologist. This mismatch often led to patients consulting several HCPs before diagnosis.

## Discussion

This study is, to our knowledge, the first to interview patients with RA quantitatively, using a bespoke structured assessment form about their initial symptoms and journey until referral. Pain was the most remembered symptom at onset and the most important reason to visit an HCP, and symptoms were mostly present in multiple joints. The GP was usually the first contacted HCP. More than one-quarter of the patients required more than five visits before RA was thought probable. The data provide insight into how patients with early RA perceive their first symptoms and how they will probably report symptoms to the HCP they visit first.

In our study, we saw that pain was the most commonly reported initial symptom of RA and the main reason to visit an HCP. These findings confirm results of previous studies [24, 26, 30–32]. Furthermore, other studies indicate that pain relief was highly preferred from the perspective of both patients with early RA or established RA [33, 34]. Interestingly, fatigue was mentioned as an initial symptom at onset in more than one in five patients, suggesting the importance of fatigue from the start of symptoms. Fatigue is indeed often considered a common symptom in RA, both in early and especially in established RA [24, 35, 36]. In contrast, a previous qualitative study conducted by Van der Elst *et al.* [34] reported that after 1 year, patients who are treated intensively assign less importance to fatigue compared with pain as a preferred outcome for the evaluation of treatment effect, contrary to the very early disease phase. Hence, the role of fatigue in the detection of RA is not yet known, indicating a lack of knowledge of the true hallmarks of RA, aside from joint involvement, could be at symptom onset.

Only about half of the GPs detected first symptoms as possibly RA related at the first visit in patients who were later diagnosed with RA. Furthermore, in 25% of our study population, more than five visits were required to make a diagnosis of probable RA, leading to longer total treatment delays. This trend, where more visits were needed in the months shortly before diagnosis, has been seen in another study [25]. A qualitative exploration by Meyfroidt *et al.* [37] showed that in Belgium GPs expressed not feeling confident enough in the detection of RA owing to the indistinct nature of early RA-related symptoms, the frequent inconclusiveness of diagnostic tests and the scarceness of RA cases in general practice. These findings might explain the multiple visits of patients to their initial HCP. Mølbæk *et al.* [31] showed that the nature and severity of symptoms, apart from the presence of other diseases, determine how the clinical picture is interpreted both by the GP and by the patient. Algorithms supporting the GP in detecting possible RA cases have been developed but need validation in daily clinical practice [38, 39].

As expected, the GP is the first HCP to be contacted, and the rheumatologist is the physician mostly referred to as the second line. Of interest, many patients indicated that their feet were affected or that they were using supportive devices. This result could explain why the orthopaedic surgeon is the second most frequently contacted HCP, as both the initial and secondary contact. A Saudi Arabian study even showed that orthopaedic surgeons were the first contacted HCP in the majority of cases, although this could be attributable to local beliefs about the roles of the GP, orthopaedic surgeon and/or rheumatologist concerning musculoskeletal problems [40]. Nevertheless, orthopaedic surgeons, apart from many other medical specialties, could be a target for awareness campaigns and postgraduate education to improve the help-seeking trajectory of many patients. Improving the RA detection skills of orthopaedic surgeons and other HCPs, but also the knowledge of the general public regarding RA remains of utmost importance to speed up the referral process of persons susceptible to RA to a rheumatologist [23]. However, it is hard to generalize the ideal targets to improve RA detection, because the health-care context differs between countries.

The additional sketches and notes showed the heterogeneous help-seeking trajectories of patients, with many different HCPs and rheumatologists involved in this process, as seen by others [24]. A long and/or complex patient trajectory, with the participation of several HCPs or rheumatologists, can have a major impact on the patient. Patients must invest time in this trajectory, often feeling insecure about the possible cause of disease and its consequences [32, 41]. These issues might possibly lead to a decreased confidence in their HCPs. On top of this, initial symptoms are reported sometimes to be episodic, and some patients felt misunderstood or not listened to by their HCP or rheumatologist, often leading to them switching HCP. These reasons could also prolong the delay in treatment. Our study shows that an opportunity for more rapid RA recognition lies in familial experience with RA, as seen elsewhere [42].

A limitation of our study is that only patients from an academic hospital were included, a population with a slightly longer treatment delay compared with other settings in Belgium [15]. Another important limitation is recall bias. The accuracy of remembering the date of RA symptom onset declines over a period of 5 years [43]. Moreover, the explorative thematic analysis was not originally planned in this study. However, because patients were assessed by study team members, clarifications could be provided during the inquiry if necessary, and these were noted systematically. These additional notes provided further detailed insight into patients’ perceptions regarding their first symptoms, their help-seeking behaviour and the referral pathway, which we considered valuable enough to integrate into our analysis. This information also underlines how patients struggled with their personal trajectory to a correct diagnosis, underlying the importance of research into treatment delay from the perspective of the patient.

The fact that we were able to explore the early disease trajectory from the patient’s perspective was also the main strength of the present study. We obtained rich information about the patients’ first symptoms and how they experienced their referral, by the systematic use of a bespoke structured assessment form, developed especially for this purpose and based on questions routinely asked in daily clinical practice.

### Conclusions

This study revealed the complexity of detection of RA and the patients’ journey from symptom onset until referral to a rheumatologist. Excess pain appears to be the most important trigger for seeking help in persons susceptible to RA. GPs seem to play a pivotal role in RA detection, but the complexity of their role is underlined by the multitude of different initial symptoms attributed to RA. Referral to a rheumatologist is therefore sometimes delayed by clinical uncertainty, leading to several GP visits and longer treatment delays. This, in turn, has an impact on the patient’s perception and health behaviour, which could later also have an adverse effect on the disease outcome.

## Supplementary Material

rkz035_Supplementary_Material
