# Supplementary material for: The perspective of patients with early rheumatoid arthritis on the journey from symptom onset until referral to a rheumatologist
Source: Rheumatol Adv Pract. 2019 Aug 30;3(2):rkz035. doi: 10.1093/rap/rkz035 (PMC7018614; doi:10.1093/rap/rkz035)
Supplement: rkz035_Supplementary_Material [file rkz035_supplementary_material.docx]

**SUPPLEMENTARY MATERIAL**

**Supplement 1: The bespoke assessment form (dutch)**

**Anamnese - delay bij Reumatoïde Artritis (RA)**

**DEEL 1: DE PATIENT**

1. **Wanneer begonnen de eerste symptomen/klachten i.v.m. RA?** *(datum)* **____________________________________________________________________________**
2. **Wat was/waren de eerste symptomen/klachten?** *(Meerdere antwoorden mogelijk.)*

| - **Pijn** | - - - **In 1 gewricht**     - **In meerdere gewrichten** | - - - **Vingers/handen/pols**     - **Tenen/voeten/enkels**     - **Knieën**     - **Schouders/ellebogen** | **Specifiek:**  **Specifiek:**  **Specifiek:**  **Specifiek:** |
| --- | --- | --- | --- |
| - - - - **Stramheid** | - - - **In 1 gewricht**     - **In meerdere gewrichten** | - - - **Vingers/handen/pols**     - **Tenen/voeten/enkels**     - **Knieën**     - **Schouders/ellebogen** | **Specifiek:**  **Specifiek:**  **Specifiek:**  **Specifiek:** |
| - - - - **Zwelling** | - - - **In 1 gewricht**     - **In meerdere gewrichten** | - - - **Vingers/handen/pols**     - **Tenen/voeten/enkels**     - **Knieën**     - **Schouders/ellebogen** | **Specifiek:**  **Specifiek:**  **Specifiek:**  **Specifiek:** |
| - **Vermoeidheid** - **Ochtendstijfheid** - **Andere:** | | | |

1. **Wanneer ontstonden de eerste musculoskeletale problemen?** *(datum)* **_________________***(Indien dit niet als eerste symptoom* (pijn, zwelling, stramheid in gewrichten) *werd gerapporteerd)*
2. **Wanneer ontstond de eerste persistente gewrichtszwelling?** *(datum)* **__________________***(Indien dit niet als eerste symptoom werd gerapporteerd)*
3. **Hebben er bepaalde gebeurtenissen plaatsgevonden juist vóór/tegelijk met de eerste opkomende RA klachten?**
   - **Blessure**
   - **Zwangerschap**
   - **Chirurgische ingreep**
   - **Infectie**
   - **Vaccinatie**
   - **Andere: _________________**
4. **Wie hebt u in eerste instantie omwille van uw RA symptomen bezocht?**

- **Huisarts**
- **Reumatoloog**
- **Kinesist**
- **Orthopedische chirurg**
- **Neuroloog**
- **Osteopaat**
- **Andere: _________________**

1. **Wanneer bent u voor uw RA symptomen naar de (huis)arts (of andere) gegaan?** *(datum)*

**____________________________________________________________________________**

1. **Waarom hebt u besloten naar uw (huis)arts te gaan?**

- **Hinder bij dagelijkse activiteiten**
- **Te hevige pijn**
- **Te hevige stramheid**
- **Opvallende gewrichtszwelling**
- **Hinder bij uitvoering van job**
- **Andere: _________________**

**DEEL 2: DE (HUIS)ARTS**

1. **Heeft uw (huis)arts bij het eerste bezoek uw symptomen erkend als mogelijks
   RA symptomen? JA / NEE**

**Indien NEE: Aan welke aandoening dacht de (huis)arts aanvankelijk?**

**________________________________________________________**

**Indien NEE: Hoe vaak bent u naar de (huis)arts teruggekeerd vooraleer de juiste diagnose werd gesteld?**

- **1**
- **2**
- **3**
- **4**
- **5**
- **>5**

1. **Welke stappen ondernam uw (huis)arts vooraleer u door te verwijzen?**

- **Bloedonderzoek**
- **Radiologisch onderzoek**
- **Pijnstillers voorschrijven**
- **Afwachtende houding**
- **Leefstijladvies**
- **Andere: _________________**

1. **Naar wie verwees uw (huis)arts u door in eerste instantie?**

- **Reumatoloog**
- **Kinesist**
- **Orthopedische chirurg**
- **Neuroloog**
- **Osteopaat**
- **Andere: _________________**

1. **Wanneer heeft uw (huis)arts u doorverwezen naar de reumatoloog?** *(datum)*

**The bespoke assessment form (English translation)**

**Patient History - delay in Rheumatoid Arthritis (RA)**

**Part 1: THE PATIENT**

**When did the first symptoms concerning RA occur?** *(date)* **____________________________________________________________________________**

**What were the first symptoms/complaints?** *(multiple answers possible)*

| - **Pain** | - - - **In 1 joint**     - **In multiple joints** | - - - **Finger/hand/ wrist**     - **Toe/foot/ankle**     - **Knees**     - **Shoulders/elbow** | **Specific:**  **Specific:**  **Specific:**  **Specific:** |
| --- | --- | --- | --- |
| - - - - **Rigidness** | - - - **In 1 joint**     - **In multiple joints** | - - - **Finger/hand/ wrist**     - **Toe/foot/ankle**     - **Knees**     - **Shoulders/Elbow** | **Specific:**  **Specific:**  **Specific:**  **Specific:** |
| - - - - **Swelling** | - - - **In 1 joint**     - **In multiple joints** | - - - **Finger/hand/ wrist**     - **Toe/foot/ankle**     - **Knees**     - **Shoulders/elbow** | **Specific:**  **Specific:**  **Specific:**  **Specific:** |
| - **Fatigue** - **Morning Stiffness** - **Other:** | | | |

**When did the first musculoskeletal symptoms occurred** *(date)* **_________________***(if as first symptom, pain, swelling nor rigidness in the joints were reported*

**When started the first persistent joint swelling?** *(date)* **__________________***(if not reported as first symptom)*

**Where there any events occurring simultaneously/just before the first RA complaints?**

- - **Injury**
  - **Pregnancy**
  - **Surgery**
  - **Infection**
  - **Vaccinatie**
  - **Andere: _________________**

**Who did u first visit regarding your complaints?**

- **General practitioner**
- **Rheumatologist**
- **Physiotherapist**
- **Orthopedic surgeon**
- **Neurologist**
- **Osteopath**
- **Other: _________________**

**When did u first visit a healthcare professional regarding your symptoms?** *(date)*

**____________________________________________________________________________**

**Why did you go to a healthcare professional?**

- **Limitations in daily activities**
- **Too high pain**
- **Too rigid**
- **Significant joint swelling**
- **Hindered execution of job**
- **Other: _________________**

**PART 2: The Healthcare Professional**

**Did your healthcare professional recognize the symptoms immediatly as RA symptoms? YES / NO**

**If NO: Which other diagnose was suspected by the healthcare professional?**

**________________________________________________________**

**If NO: How many times did you need to visit the healthcare professional again before a suspicion of RA was made?**

- **1**
- **2**
- **3**
- **4**
- **5**
- **>5**

**Which steps did your healthcare professional do before referral?**

- **Blood taken**
- **Radiology**
- **Prescription pain medication**
- **Wait-and-see approach**
- **Advice on lifestyle**
- **Other: _________________**

**To whom were you initially referred by your healthcare professional?**

- **Reumatologist**
- **Physiotherapist**
- **Orthopedic surgeon**
- **Neurologist**
- **Osteopath**
- **Other: _________________**

**When were you referred by your healthcare professional to a rheumatologist?** *(date)*

**_________________________________________________________________________**
